# 360 Degrees of Facial Perception: Congruence in Perception of Frontal Portrait, Profile, and Rotation Photographs

**DOI:** 10.3389/fpsyg.2018.02405

**Published:** 2018-12-07

**Authors:** Vít Třebický, Jitka Fialová, David Stella, Zuzana Štěrbová, Karel Kleisner, Jan Havlíček

**Affiliations:** ^1^National Institute of Mental Health, Klecany, Czechia; ^2^Faculty of Science, Charles University, Prague, Czechia

**Keywords:** 2D, 3D, head, standardized photography, assessment, morphology, attractiveness, formidability

## Abstract

Studies in social perception traditionally use as stimuli frontal portrait photographs. It turns out, however, that 2D frontal depiction may not fully capture the entire morphological diversity of facial features. Recently, 3D images started to become increasingly popular, but whether their perception differs from the perception of 2D has not been systematically studied as yet. Here we investigated congruence in the perception of portrait, left profile, and 360° rotation photographs. The photographs were obtained from 45 male athletes under standardized conditions. In two separate studies, each set of images was rated for formidability (portraits by 62, profiles by 60, and 360° rotations by 94 raters) and attractiveness (portraits by 195, profiles by 176, and 360° rotations by 150 raters) on a 7-point scale. The ratings of the stimuli types were highly intercorrelated (for formidability all rs > 0.8, for attractiveness all rs > 0.7). Moreover, we found no differences in the mean ratings between the three types of stimuli, neither in formidability, nor in attractiveness. Overall, our results clearly suggest that different facial views convey highly overlapping information about structural facial elements of an individual. They lead to congruent assessments of formidability and attractiveness, and a single angle view seems sufficient for face perception research.

## Introduction

When artists create portraits, they rarely depict a full frontal view of the face of a given sitter. Instead, they tend to portray people in some degree of profile, emphasizing one cheek and dimensionality of a face (Murphy, 1994). Interestingly, vast majority of studies on facial perception uses frontal portraits (un/altered photographs, morphs, or line drawings) as stimuli (e.g., Thornhill and Gangestad, 1999; Rhodes, 2006; Kościnski, 2009; Calder et al., 2011; Little et al., 2011; Valentová et al., 2013; Little, 2014). Given, however, that in our daily lives we experience faces from multiple angles, it is far from certain that a frontal view is the optimal depiction and several studies even suggested that an individual's appearance can significantly vary depending on the viewing angle (Rule et al., 2009; Jenkins et al., 2011; Tigue et al., 2012; Kościnski and Zalewska, 2017; Sutherland et al., 2017). Faces are, after all, complex and highly variable morphological structures (Enlow et al., 1996) and some facial features are apparent only from some viewing angles (Danel et al., 2018). For example, Danel et al. (2018) reported only a moderate correlation in sexually dimorphic features between lateral and frontal facial configuration in both men and women. When frontal and lateral facial configurations were compared as to their averageness, a significant association was found only in women. It is therefore plausible to assume that complementary information may be provided by different viewing angles. A single frontal view could potentially obscure relevant visual cues used in assessing certain dimensions (e.g., determinants of facial masculinity, such as protrusion of the brow ridge and angularity of the jaw), thus reducing judgment accuracy.

In research on body perception, the use of other than just frontal view is becoming increasingly common (Tovée and Cornelissen, 2001; Perilloux et al., 2012; Sell et al., 2017; Cornelissen et al., 2018). The use of multiple body angles views allows for assessments of multivariate trait interactions (Brooks et al., 2015). Varying viewing angles of bodies allow raters to assess the shapes and sizes of various morphological characteristics, such as body fat, lean mass distribution, or breast morphology (Dixson et al., 2011, 2015) which all contribute to the resulting attractiveness rating.

Research on facial perception that employs other than frontal facial views remains, however, at best unsystematic (Kościnski, 2009) and mutual relations between the frontal and lateral dimensions of facial features have so far received very little attention (Danel et al., 2018). Profile views have been used primarily in orthodontics and aesthetic medicine because it is known that they have an impact on facial attractiveness judgments (Spyropoulos and Halazonetis, 2001; Johnston et al., 2005; Maple et al., 2005; Soh et al., 2007; Shafiee et al., 2008; Nomura et al., 2009). Results from several studies that investigated the averageness of facial profiles show patterns analogous to frontal images (Spyropoulos and Halazonetis, 2001; Minear and Park, 2004; Valentine et al., 2004; Valenzano et al., 2006). Some researchers, meanwhile, tried to overcome the limitations of a single view stimulus by presenting raters with both frontal and lateral views of targets on a single screen (e.g., Dixson and Rantala, 2015; Dixson et al., 2016; Valentova et al., 2017), while other studies found a medium to high correlation between the rating of attractiveness of frontal and lateral depictions (ranging from r = 0.52 to 0.83) (Diener et al., 1995; Valenzano et al., 2006; Davidenko, 2007; Shafiee et al., 2008; Kościnski and Zalewska, 2017).

Until recently, most studies used as stimuli static, two-dimensional images (photographs). Thanks to technological progress, including a considerable increase in computers' computing powers, 3D scanning and 3D reconstruction technology is now becoming more accessible to facial perception research (Toole et al., 1999; Caharel et al., 2009; Chelnokova and Laeng, 2011; Meyer-Marcotty et al., 2011; Jones et al., 2012; Lefevre et al., 2012; Tigue et al., 2012; Berssenbrügge et al., 2014; Mydlová et al., 2015; Holzleitner and Perrett, 2016; Hu et al., 2017; Kordsmeyer et al., 2018). Potential bias associated with a single 2D image (e.g., profile) might be minimized by the use of 3D images, which represent various viewing angles. To our best knowledge, however, only one study directly compared ratings based on 2D and 3D facial images (Tigue et al., 2012). Authors found a high correlation between 2D and 3D stimuli (*r* = 0.71), with mean ratings significantly higher for 3D images. On the other hand, it should be noted that in this study, only opposite-sex ratings were performed (female faces were rated by male participants), on a single scale (attractiveness), and the only 2D depictions used were frontal portraits.

Current evidence suggests a rather high level of congruence in judged characteristics (especially attractiveness) between frontal and lateral or frontal and 3D views of faces. It should, however, be taken into account that the development of morphological features between the frontal and lateral view does not always correlate (Danel et al., 2018) and one could thus expect that some socially relevant traits may be easier to assess from other than frontal view (Tigue et al., 2012).

In the two studies, we estimated the congruence in perception of three different views of male heads (frontal portrait, left profile, and 360° rotation photographs). We employed two characteristics relevant in the context of intra- and inter-sexual selection, namely the rating of formidability and attractiveness. We also explored whether the type of device used (mobile phones, laptop, and desktop computers) influences the ratings.

## Materials and Methods

All procedures employed in this study conform to the ethical standards of the relevant committee on human experimentation and with the Helsinki Declaration. The study was approved by the Institutional Review Board of National Institute of Mental Health, Czech Republic (Ref. num. 28/15). All participants were informed about the goals of the study and gave their informed consent. The present study is part of a larger project which investigates multimodal perception of traits associated with sexual selection and characteristics related to competition outcome.

### Targets

We collected photographs of 45 male Mixed Martial Arts (MMA) athletes (mean age = 26.6, *SD* = 5.86, range = 18–38). All athletes were from the Czech Republic. They were invited via social media advertisements, leaflets distributed at domestic MMA tournaments, gyms, and with the assistance of Mixed Martial Arts Association Czech Republic (MMAA). All targets were provided with brief description of the project and approved their participation by signing informed consent. As compensation for their participation, they received 400 CZK (approx. €15).

### Acquisition and Settings of the Photographs

To capture images of the targets' head from all 360°, we built a turning plywood platform (120 cm in diameter) using flat ball bearings. The platform had 36 steps around its perimeter, i.e., one step for every 10°, making it basically a large turntable. To achieve standardization—all photographs were acquired on site—the platform was placed inside a purpose-built portable photographic booth to control for changes in ambient illumination and for color reflections (see e.g., Rowland and Burriss, 2017; Thorstenson, 2018). Booth dimensions were 140 × 140 × 255 cm. Its frame was made of sectioned aluminum profiles. The outside of the booth and the inside of its roof was covered with black duvetyn cloth (a dense fabric), while the internal side of the walls, the seamless backdrop, and surface of the turning platform were covered with a bright white velvet (medium density fabric).

To achieve standardized lighting conditions, we used one 800 W studio strobe (Photon Europe MSN HSS-800) aimed into a white reflective umbrella used as a light modifier (Photon Europe, 109 cm diameter), mounted on a 175 cm high light stand, and tilted 10° downwards toward the booth. The light was positioned 125 cm from the target. This lighting setup ensured even exposure across the whole scene, which was further verified before each session by a digital light meter (Sekonic L-308S).

Images were acquired using a 24-megapixel, full-frame (35.9 × 24 mm CMOS sensor, a 35 mm film equivalent) digital SLR camera Nikon D610 equipped with a fixed focal length lens Nikon AF-S NIKKOR 85 mm f/1.8 G (Třebický et al., 2016). Exposure values were set to ISO 100, shutter speed 1/200 s, and aperture f/11. Photographs were shot into 14-bit uncompressed raw files (NEF) and AdobeRGB color space. Color calibration was performed using X-Rite Color Checker Passport color targets and white balance patch photographed at the beginning of each session. The camera was mounted in portrait orientation directly onto the light stand, which carried also the strobe light positioned 125 cm from the target so as to achieve a perception close to social interpersonal distance (Hall, 1966; Baldassare and Feller, 1975; Sorokowska et al., 2017), to maintain a constant perspective distortion (Třebický et al., 2016; Erkelens, 2018), and to avoid potential perception bias based on interpersonal distance (Bryan et al., 2012). Camera's distance from each target was verified with a digital laser rangefinder (Bosch PLR 15) as distance between the sensor plane (marked ϕ on camera body) to the middle of target's forehead. Camera's height was adjusted for each target so as to position the center of his head in the middle of the frame. Focus point was set on target's right eye and focus distance was locked for further images of the target. This setting of camera's distance, focal length, and sensor size yielded a 35 × 53 cm field of view (23.85° angle of view) and the aperture setting resulted in a 9 cm depth of field (4 cm before and 5 cm behind the focal plane).

Targets were seated on a 63 cm high bar stool (Ikea Franklin) positioned in the middle (rotation axis) of the turning platform. We asked them not to lean against the stool's back support and to sit with their back straight and hands hanging freely alongside their body. They were asked to adopt a neutral facial expression (with no smile or frown), to look directly into the camera, and to remain in this position for all subsequent photographs. When necessary, targets were instructed to adjust their posture and head position, so they were facing the camera straight on, without any head pitch, yaw, or roll. On top of that, they were instructed to wear only black underwear shorts we provided them with (i.e., without T-shirt) and to remove any adornments (glasses, earrings, piercings or other jewelry).

One full 360° rotation yielded 36 photographs. After each photograph, research assistant manually turned the turning platform by one step (10°) clockwise. We captured two full rotations to obtain one full set of images while eliminating all possible movements, blinks, etc. of targets. Capturing both full rotations took approx. 10 min.

### Stimuli Processing

All image processing was carried out in Adobe Lightroom Classic CC (Version 2017). First, all images were converted into DNG raw files and DNG color calibration profiles were assembled (using X-Rite Color Checker Passport LR plugin) and applied to all photographs. For each target, a final set of 36 images covering full 360° head rotation was selected and postprocessed by combining suitable images (correct head position, open eyes, closed mouth, etc.) from the two captured rotations. To ensure consistency in exposure across all selected photographs, percentages of Red, Green, and Blue channel values were checked across three background areas (above, left, right) and eventual small differences in exposure were manually adjusted to the same level. In the next step, the calibrated images were exported into lossless 16-bit AdobeRGB TIFF files in their real size of 35 × 53 cm and 168 pixels per inch (ppi) resolution (a native ppi of 4K screens used for rating sessions, see Rating Session in section Formidability Rating). This resulted in life-sized images of targets' heads. Horizontal and vertical positions of images were adjusted using LR Transform tool to position target's head in the center of the frame with eyes in a horizontal line. Final images were batch-cropped to 1:1.1 (2,095 × 2,305) side ratio to fit head rotations of all targets. Images were then converted into sRGB color space and exported as 8-bit JPEG files (2,095 × 2,305 px @ 168 ppi).

#### Building 360° Head Rotations

We used Sirv (www.sirv.com), an online suite for creating and managing image spins, to build 360° head rotations. With all image adjustments and optimization to image size and quality done by Sirv turned off, we uploaded the images of all targets and created the individual spins. See Supplementary Materials for sample 360° head rotation (360 rotation video.MP4).

#### Portraits and Profiles

Analogously to previous research investigating morphological differences between portraits and profiles (Danel et al., 2018), we have selected from the set of 36 images for each target a frontal and left profile image. See Supplementary Materials for sample frontal portrait (Frontal portrait.JPEG) and left profile (Left profile.JPEG).

### Raters

#### Formidability Rating

Portraits were evaluated by 62 raters (30 men), mean age = 23.1 (*SD* = 3.45, range = 18–39); profiles by 60 raters (30 men), mean age = 22.8 (*SD* = 3.55, range = 18–36); and 360° rotations by 94 raters (46 men), mean age = 22.1 (*SD* = 3.09, range = 18–38) (see **Table 2**). Raters were mainly Charles University (Prague, Czech Republic) students recruited via social media advertisements, mailing list of participants assembled in previous studies or invited on site. All raters were provided with brief description of the project and approved their participation by signing informed consent. Rating took place in a lab (see section Rating Sessions, Formidability Rating) and when the rating was completed, they received for their participation 100 CZK (approx. €4) and a debriefing leaflet. Using a two-way ANOVA, we found no age differences between sexes, stimuli type ratings, or their interaction [Sex: *F*_(1, 210)_ = 0.371, *p* = 0.543; Stimuli type: *F*_(2, 210)_ = 1.777, *p* = 0.172; Sex × Stimuli type: *F*_(2, 210)_ = 0.006, *p* = 0.994].

#### Attractiveness Rating

Portraits were evaluated by 195 raters (30 men), mean age = 29.6 (*SD* = 6.05, range = 18–48); profiles by 176 raters (32 men), mean age = 29.2 (*SD* = 6.26, range = 18–53); and 360° rotations by 150 raters (35 men), mean age = 29 (*SD* = 6.27, range = 18–46) (see **Table 2**). Raters were recruited mainly via advertisements among followers of National Institute of Mental Health (facebook.com/nudzcz) and Human Ethology group (facebook.com/etologie) Facebook pages. Ratings were carried out online. All raters provided their informed consent by clicking on the “I agree” button to consent with their participation in the study and were not financially reimbursed. Two-way ANOVA showed no age difference between sexes, the stimuli type ratings or their interaction [Sex: *F*_(1, 515)_ = 2.553, *p* = 0.111; Stimuli type: *F*_(2, 515)_ = 0.162, *p* = 0.85; Sex × Stimuli type: *F*_(2, 515)_ = 0.084, *p* = 0.864]. **Table 2** provides detailed descriptive statistics.

### Rating Sessions

#### Formidability Rating

Formidability ratings were performed in two separate sessions. In the first session, we collected the ratings of 360° rotations. In the second session, raters were randomly divided to rate either a set of portrait or profile images. Each rater thus judged a full set of only one type of stimuli.

Ratings took place in a quiet perception lab, in standardized conditions across all raters (with artificial lighting and closed window blinds to eliminate changes in ambient lighting). Raters were seated in the same eye level with stimuli's eyes, 125 cm from the screen, i.e., in the same distance as the camera was from the target in order to simulate approximate social interpersonal distance (Hall, 1966; Baldassare and Feller, 1975), and in the center of the projected photograph (Cooper et al., 2012). This was implemented so as to increase the ecological validity of the rating.

Images were presented to raters on 27′′ Dell U2718Q UltraSharp IPS screens (3,840 × 2,160, 99% sRGB color space coverage) turned to a vertical position to accommodate life-size images. Screens were connected to Asus ROG G20 PC running Microsoft Windows 10 with environment scaling set to 100%. Screens were color- and luminance-calibrated with X-Rite i1 Display Pro probes. The probes were connected during the whole rating session to adjust screens for ambient light. Qualtrics survey suite (Qualtrics, Provo, UT) with Blank theme run through Google Chrome (in full screen mode and 100% scaling) was used for data collection.

All raters received a set of brief demographics questions (e.g., sex, age, and education status) followed by a block containing stimuli. Images were presented in a randomized order. Raters were asked to rate formidability (“Jak moc by byl tento muž úspěšný, kdyby se dostal do fyzického souboje?”/“If this man was involved in physical confrontation, how successful he would be?”) of each target on a 7-point verbally anchored scale (from “1 – velice neúspěšný”/“very unsuccessful,” to “7 – velice úspěšný”/“very successful”). The 360° rotations spun automatically clockwise once (automatic rotation took approx. 2 s) and raters were instructed to turn the heads around for further inspection by dragging mouse left or right before rating. Portrait and profile photographs were simply projected on the screen. Time for rating was not limited.

#### Attractiveness Rating

Ratings were collected on-line via Qualtrics survey suite (Qualtrics, Provo, UT). All raters were first presented with a brief study description and informed consent. Then they completed a set of demographics questions, which was followed by one randomly selected block of stimuli (portraits, profiles, or 360° rotations). Each rater thus assessed a full set of only one type of stimuli. Images were presented in a randomized order. Raters were asked to rate attractiveness (“Jak atraktivní je muž na fotografii?”/“How attractive is the man on photograph?”) of each target on a 7-point verbally anchored scale (from “1 – velice neatraktivní”/“very unattractive”, to “7 – velice atraktivní”/“very attractive”). The 360° rotations spun automatically once clockwise (automatic rotation took approx. 2 s) and raters were instructed to turn the heads around for further inspection by dragging mouse left or right before rating, while portrait and profile photographs were simply projected on the screen. Time for rating was not limited.

We used Qualtrics *Blank theme* and custom CSS code to set the image size to 800 px width with centered margin alignments (*.Skin #SkinContent.QuestionBody {width: 800px; display: block; margin-left: auto; margin-right: auto;}.Skin #SkinContent.QuestionText {width: 800px; display: block margin-left: auto; margin-right: auto;}*) to standardize stimulus size and position across all devices used. First, raters were asked to switch their web browsers into Full Screen mode and adjust page scaling to achieve the largest image size possible while seeing the rating scale without having to scroll down, i.e., if a Full HD 16:9 screen (1,920 × 1,080) was used for rating in Full Screen mode, browser scaling would remain on native 100%.

##### Devices used for attractiveness rating

When raters completed rating the images, they were asked to specify the type of device they used (mobile phone, tablet device, laptop computer, desktop computer or other), screen size or brand and model name of the device (to later identify screen size and resolution). This data was used to test a possible effect of the device used on the rating.

In total, attractiveness was rated by 521 raters: 233 used laptop computers, 135 desktop computers, 116 mobile phones, 19 tablet devices, 1 other device, and 17 did not specify device type. See Tables S1 and S2 for data on screen sizes and resolutions.

In subsequent analyses, we used only data from the three most frequently represented device categories: mobile phones, laptops, and desktop computers. This resulted in a sample of 484 raters.

### Statistical Analysis

All statistical tests were performed in JASP 0.9.0.1 (JASP Team, 2018) and jamovi 0.9.1.7 (jamovi project, 2018). McDonald's ω statistics was used for estimating inter-rater agreement (Dunn et al., 2014). To test for potential age differences between rater groups, a two-way ANOVA was carried out, with raters' sex and stimuli types entered as two independent variables and age as a dependent variable. Two-way ANOVA was further used to compare sex differences in mean formidability and attractiveness rating, where raters' sex and stimuli types were entered as two independent variables and the rating of formidability or attractiveness as a dependent variable. Effect sizes for two-way ANOVAs are reported in η^2^. Associations between the ratings of different stimuli types were tested by bivariate correlations using Pearson's r coefficient with 95% CIs [lower limit, upper limit]. For exploratory purposes, we also tested the influence of device on attractiveness rating using a two-way ANOVA with stimulus type and device type entered as independent variables and mean attractiveness rating as a dependent variable. A Holm's *post-hoc* test was performed and effect sizes for the comparison are reported in Cohen's d.

### Data Availability

Datasets generated and analyzed during the current study are available as Supplementary Material of this article (Dataset formidability.XLSX, Dataset attractiveness.XLSX).

## Results

### Formidability Rating

McDonald's ω scores of male and female ratings showed a high inter-rater agreement across all three stimuli types (ranging from 0.732 to 0.876). In subsequent analyses, we have therefore used mean formidability ratings given to the individual stimuli separately by male and female raters. Further, we found a high correlation between ratings assigned by men and women for portraits (*r* = 0.941, 95% CI [0.895, 0.967], *p* < 0.001), profiles (*r* = 0.962, 95% CI [0.931, 0.979], *p* < 0.001) and 360° rotations (*r* = 0.972, 95% CI [0.95, 0.985], *p* < 0.001).

**Figure 1:**
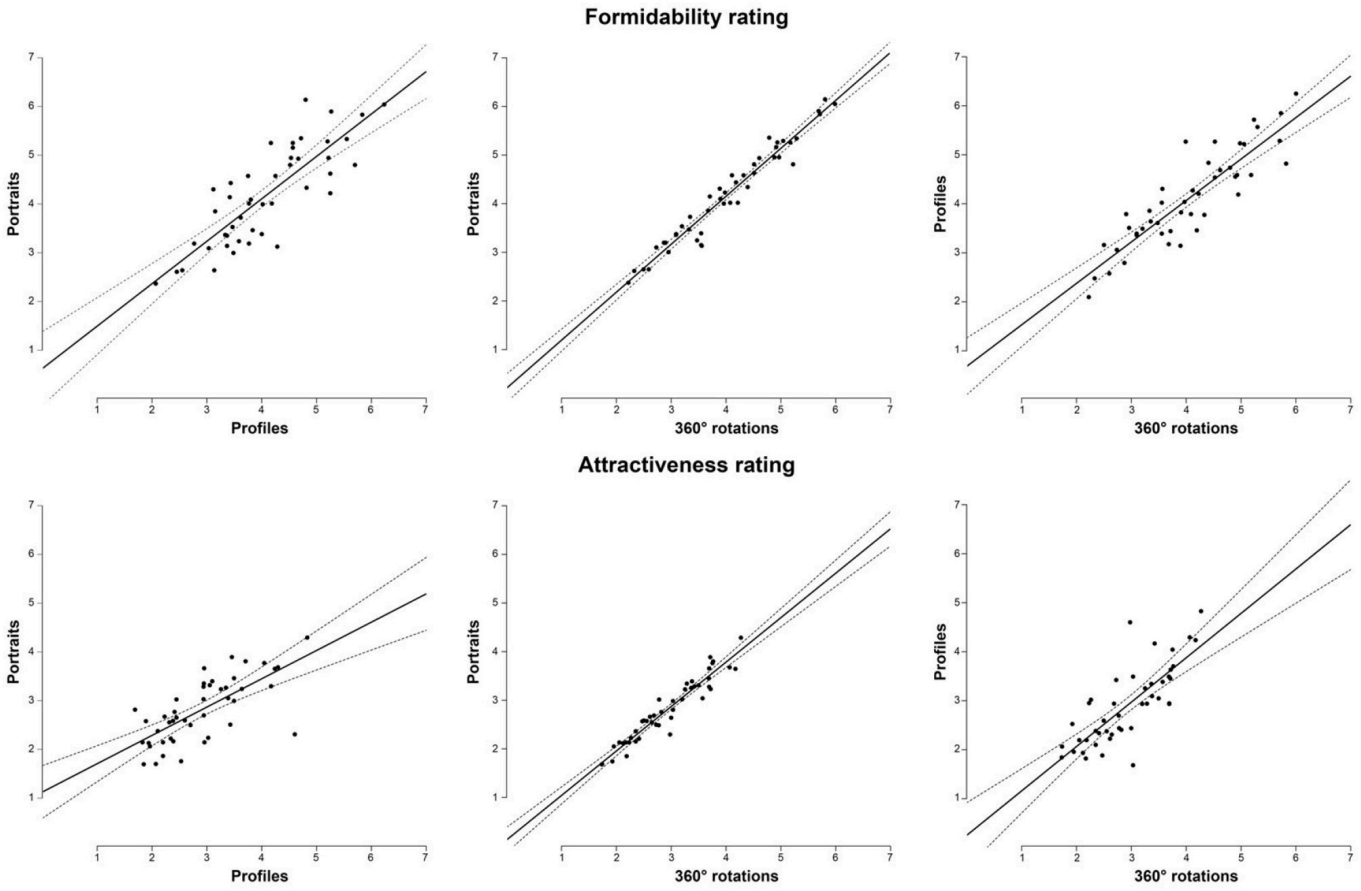
Correlations between portraits, profiles, and 360° rotations in perceived formidability (upper line) and attractiveness (lower line). Dashed lines indicate 95% CI.

**Figure 2:**
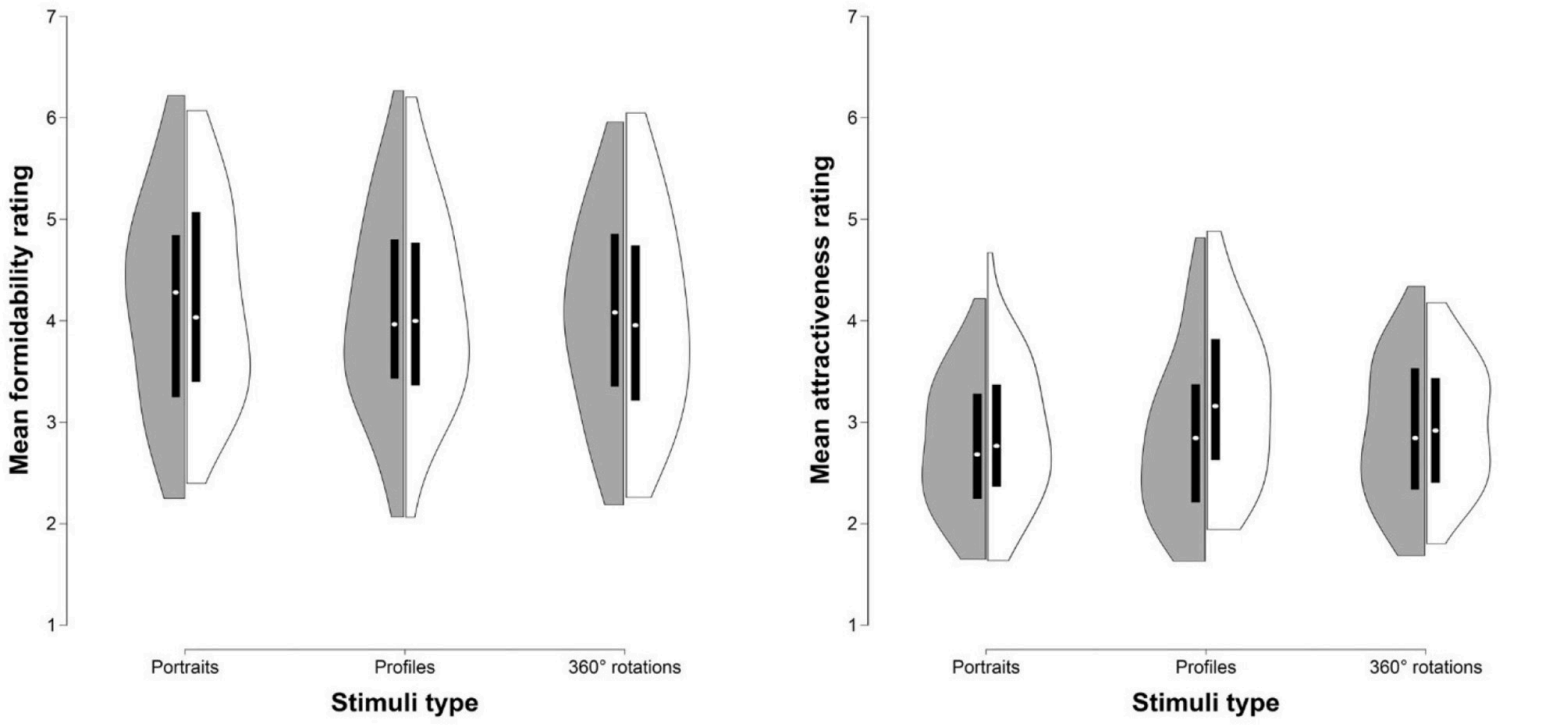
Differences in mean ratings of formidability **(Left)** and attractiveness **(Right)** between stimuli types (portraits, profiles, and 360° rotations). Violin plots show rating distributions, box plots its 25th and 75th percentile. Dark gray violin plots represent female and white violin plots male ratings, respectively. Mean formidability ratings did not differ between sexes, while males rated all stimuli types as more attractive compared to females.

Ratings of all three stimuli types were highly correlated (Table 1, Figure 1). Two-way ANOVA showed no main effect of rater's sex [*F*_(1, 264)_ = 0.00014, *p* = 0.991, η^2^ < 0.001], stimulus type [*F*_(2, 264)_ = 0.473, *p* = 0.624, η^2^ = 0.004], or rater's sex × stimulus type interaction [*F*_(2, 264)_ = 0.01, *p* = 0.99, η^2^ < 0.001] on formidability ratings (Figure 2). For descriptive statistics, see Table 2.

**Table 1:** Correlations between stimuli types.

**Scale**	**Stimuli**	**Pearson's *r* [95 % CI]**
Formidability	Portrait – Profile	0.829 [0.708, 0.903]
	Portrait – 360° rotation	0.974 [0.952, 0.985]
	Profile – 360° rotation	0.882 [0.794, 0.934]
Attractiveness	Portrait – Profile	0.706 [0.520, 0.828]
	Portrait – 360° rotation	0.956 [0.921, 0.976]
	Profile – 360° rotation	0.782 [0.634, 0.875]

*All correlations are significant at p < 0.001*.

**Table 2:** Descriptive statistics.

**Scale**	**Stimuli**	**Sex**	***N***	**Age**			**Rating**			**McDonald's ω**
				**Mean**	***SD***	**Range**	**Mean**	***SD***	**Range**	
Formidability	Portraits	Total	62	23.113	3.448	18–39	4.186	1.009	2.25–6.22	0.815
		Female	32	23.219	3.933	18–39	4.193	1.068	2.25–6.22	0.847
		Male	30	23	3.006	20–31	4.179	0.977	2.4–6.07	0.732
	Profiles	Total	60	22.816	3.553	18–36	4.085	0.961	2.07–6.27	0.856
		Female	30	22.967	3.222	19–32	4.073	0.994	2.07–6.27	0.876
		Male	30	22.667	3.907	18–36	4.098	0.946	2.07–6.2	0.844
	360° rotations	Total	94	22.127	3.094	18–38	4.046	0.998	2.19–6.04	0.798
		Female	48	21.854	4.699	18–38	4.049	0.988	2.19–5.96	0.736
		Male	46	21.957	2.556	19–29	4.042	1.022	2.26–6.04	0.857
Attractiveness	Portraits	Total	195	29.6	6.048	18–48	2.803	0.656	1.63–4.67	0.892
		Females	165	29.491	5.854	18–48	2.79	0.654	1.65–4.22	0.9
		Males	30	30.2	7.107	18–41	2.876	0.696	1.63–4.67	0.831
	Profiles	Total	176	29.188	6.257	18–53	2.904	0.796	1.63–4.87	0.962
		Female	144	28.951	6.1	18–46	2.84	0.803	1.63–4.82	0.96
		Male	32	30.25	6.924	18–53	3.194	0.784	1.94–4.87	0.964
	360° rotations	Total	150	29	6.271	18–46	2.926	0.688	1.69–4.34	0.957
		Female	115	28.687	6.353	18–46	2.909	0.715	1.69–4.34	0.954
		Male	35	30.029	5.968	20–43	2.981	0.616	1.8–4.17	0.966

### Attractiveness Rating

McDonald's ω scores of male and female ratings showed a high inter-rater agreement in all three stimuli types (ranging from 0.831 to 0.966), which is why in subsequent analyses, we used the mean formidability ratings given to a particular stimulus separately by male and female raters. Ratings by women and men were highly correlated: *r* = 0.952, 95% CI [0.915, 0.974], *p* < 0.001; *r* = 0.969, 95% CI [0.944, 0.983], *p* < 0.001; *r* = 0.962, 95% CI [0.932, 0.979], *p* < 0.001 for portraits, profiles, and 360° rotations, respectively.

Attractiveness ratings of all three stimuli types were highly correlated (Table 1, Figure 1). Two-way ANOVA showed main effect of rater's sex [*F*_(1, 264)_ = 3.87, *p* = 0.05, η^2^ = 0.014], men rated attractiveness higher as compared to women, but the effect of stimulus type [*F*_(2, 264)_ = 1.516, *p* = 0.222, η^2^ = 0.011], and rater's sex × stimuli interaction [*F*_(2, 264)_ = 1.118, *p* = 0.329, η^2^ = 0.008] on attractiveness ratings was not significant (Figure 2). For descriptive statistics, see Table 2.

#### Influence of Device Type on Attractiveness Rating

**Figure 3:**
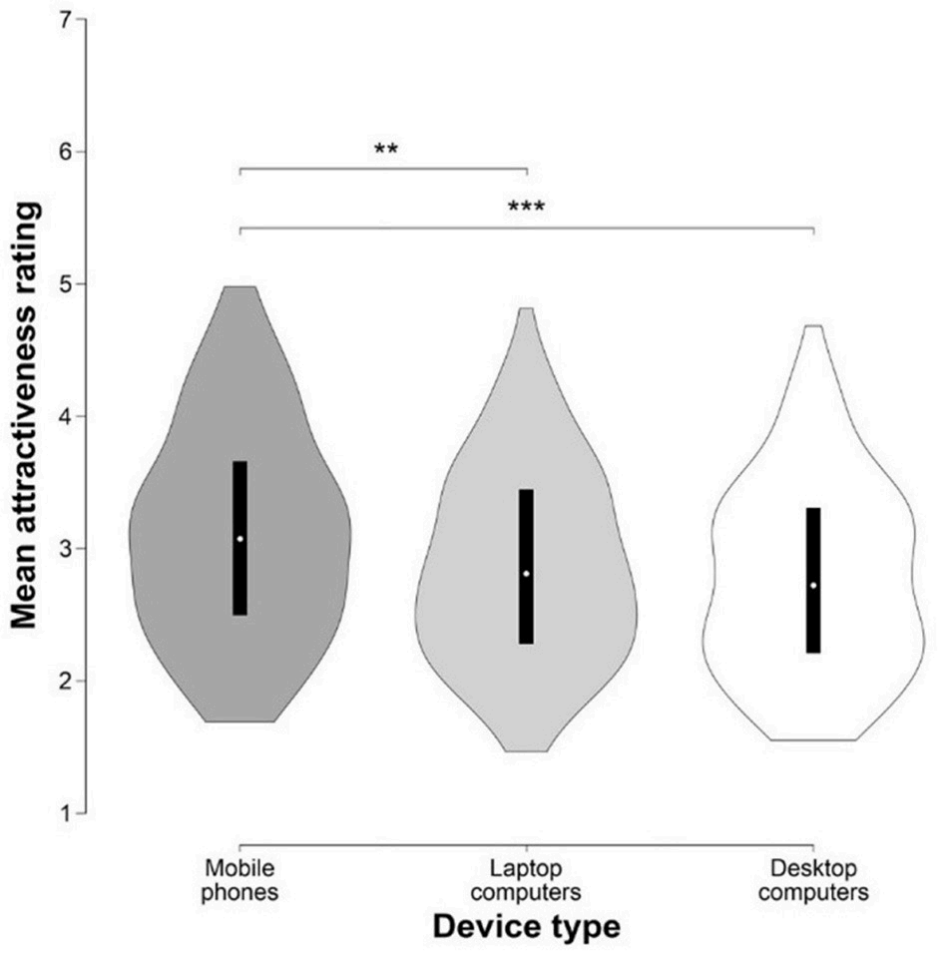
Differences in the mean ratings of attractiveness between device types. Violin plots show rating distributions, box plots its 25th and 75th percentiles. Dark gray violin plot represents mobile phones, light gray violin plot laptop computers, and white violin plot desktop computers, respectively. Asterisks indicate the level of significance, **p = 0.005, ***p < 0.001.

**Figure 4:**
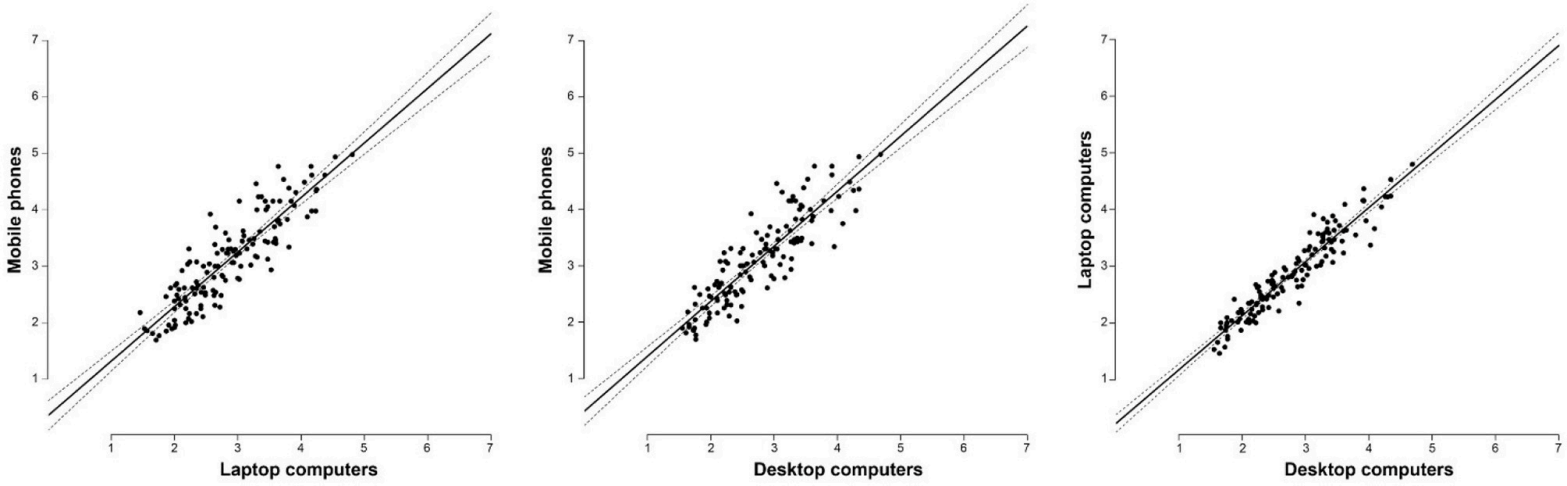
Correlations of attractiveness ratings between device types (mobile phones, laptop computers and desktop computers). Dashed lines indicate 95% CI.

To explore whether the type of device used for viewing and rating influences attractiveness rating, we performed a two-way ANOVA with stimuli type and device type as independent factors. The results showed main effects of both device types [*F*_(2, 475)_ = 7.429, *p* < 0.001, η^2^ = 0.030] and stimuli types [*F*_(2, 475)_ = 4.27, *p* = 0.015, η^2^ = 0.017], but no significant interaction between them [*F*_(4, 475)_ = 1.065, *p* = 0.373, η^2^ = 0.008]. Holm's *post-hoc* comparison showed that raters using mobile phones rated the images as significantly more attractive compared to desktop [*t*_(475)_ = 3.817, *p*_Holm_ < 0.001, Cohen's d = 0.557] and laptop users [*t*_(475)_ = 3.023, *p*_Holm_ = 0.005, Cohen's d = 0.392], whereby the ratings assigned by laptop and desktop users did not differ [*t*_(475)_ = 1.357, *p*_Holm_ = 0.175, Cohen's d = 0.145]. 360° rotations were rated significantly higher than portraits [*t*_(475)_ = 2.912, *p*_Holm_ = 0.011, Cohen's *d* = 0.418], but there was no statistical difference between 360° rotations and profiles [*t*_(475)_ = 1.753, *p*_Holm_ = 0.16, Cohen's d = 0.212]; and between portraits and profiles [*t*_(475)_ = 1.366, *p*_Holm_ = 0.173, Cohen's d = 0.159] (Figure 3). For descriptive statistics, see Table 3. Further, attractiveness ratings between all three types of devices were highly correlated: *r* = 0.883, 95% CI [0.839, 0.915], *p* < 0.001; *r* = 0.885, 95% CI [0.842, 0.917], *p* < 0.001; *r* = 0.949, 95% CI [0.93, 0.964], *p* < 0.001 for mobile phones–laptops, mobile phones–desktops, and laptops–desktops, respectively (Figure 4).

**Table 3:** Devices and rating descriptive statistics.

		***N***	**Mean attractiveness rating**	***SD***
Device	Mobile phone	116	3.021	0.625
	Laptop computer	232	2.876	0.668
	Desktop computer	136	2.777	0.634

## Discussion

The main aim of this study was to examine whether perception of formidability and attractiveness varies depending on the angle under which a face is viewed. To this purpose, we used standardized sets of frontal portraits, left profiles, and 360° head rotations of male facial images. We found strong correlations between the three types of stimuli and no significant differences in the mean ratings of formidability and attractiveness. Our results thus showed that ratings based on the three different face views were highly congruent and both perceived formidability and attractiveness ratings appear to be view-invariant. As a subsidiary aim, we have also tested the effect of the device used on attractiveness rating. While there were no differences between ratings performed on desktop and laptop computers, ratings performed using the mobile phones were higher (targets were perceived as more attractive).

Majority of facial perception research uses as stimuli frontal images (Kościnski, 2009), which is in striking contrast with our daily life experience. Moreover, there is a long-standing debate on how human visual system recognizes objects viewed from different angles (Hayward, 2003) and whether object recognition is view-specific, i.e., linked to a specific viewing orientation (Tarr and Bülthoff, 1995), or view-invariant (Biederman and Gerhardstein, 1993). Some evidence suggests that human visual system may be view-specific and process objects differently depending on the viewing angle (Jeffery et al., 2007; but see Jiang et al., 2006). If this were the case, results from perceptual studies that rely solely on frontal portraits could not be generalized to the other viewpoints. Our results, however, at least when it comes to social perception, do not support this hypothesis.

Our data shows patterns analogous in direction and magnitude to those reported in previous studies that compared assessments based on different stimuli views of both faces and bodies (e.g., frontal × profile, frontal × 3D or oblique poses), which likewise showed strong correlations in ratings (Diener et al., 1995; Tovée and Cornelissen, 2001; Valenzano et al., 2006; Davidenko, 2007; Shafiee et al., 2008; Perilloux et al., 2012; Tigue et al., 2012; Dixson et al., 2015; Kościnski and Zalewska, 2017). A related study by Tigue et al. (2012) reported that attractiveness of frontal and 3D depictions of women's faces as rated by men were highly correlated (*r* = 0.71) but 3D stimuli received significantly higher mean ratings. Authors suggest that their findings may be an effect of novelty of the 3D visualization. Our study, on the other hand, found no differences between the mean ratings of 2D (frontal and profile) and 3D images. It should be noted, however, that we opted for an alternative to standard 3D visualization. By combining several individual photographs presented in sequence as a spin (360° rotation), we avoided possible bias based simply on differences in capture technology (such as noticeable differences in lighting and colors between 2D and 3D stimuli).

The 360° rotation photographs allowed us to present raters with an all-around view of stimuli heads without running the cost of acquiring 3D capture technologies. Although the resulting visualizations are indeed photorealistic, there is a notable drawback related to implementing this procedure. The capturing and subsequent processing of the images is rather time-consuming and physically demanding, especially for the photographed targets, because one spin takes approx. 5 min and during this time, targets have to sit completely still with fixed gaze, so that controlling for head tilts, yawns, and rolls thus becomes even more critical (Penton-Voak et al., 2001; Hehman et al., 2013; Sulikowski et al., 2015). The use of 3D stimuli captured with actual 3D scanning and 3D reconstructions technology would allow for a variety of target applications including stimuli capture and presentation. For instance, resulting models could be rotated to arbitrary angles relative to their position during capture, rather than simply displayed in an identical head position in all photographs. Such 3D stimuli would produce more realistic face reconstructions: the main obstacle is the relatively high initial investment into a 3D scanner. Moreover, although 3D facial models are remarkably human-like, they are certainly distinguishable from, and less familiar than, photographs and that could potentially reduce their validity in terms of being a realistic visualization of humans (Crookes et al., 2015). Future studies should investigate whether perception of 3D models differs from 360° rotation photographs.

Interestingly, we found that the device used for viewing the stimuli has a significant influence on the rating. Raters using mobile phones gave on average higher attractiveness ratings than users of laptop or desktop computers. To our best knowledge, no previous study investigated the influence of the device used for viewing on ratings. Although the screen size and resolution of mobile phones are increasing, screen size of handheld devices does limit the size of images that can be viewed on it. That negatively influences the amount of detailed visual information available to the observer, hence potentially limiting the visibility of cues that may affect some aspects of social perception (such as attractiveness). All this may result in ratings higher than those based on viewing images on larger screens which do show more detail. For instance, several studies have reported that more homogenous skin is perceived as more attractive (Jones et al., 2004; Fink et al., 2006; Tsankova and Kappas, 2016; Jaeger et al., 2018; Tan et al., 2018). It is thus possible that lower visibility of such types of imperfections on mobile phones may lead to higher scores. This is a potentially important issue since ever more researchers opt for online data collection. One ought to take into consideration the kind of devices raters decide to use for their viewing and rating, because if a specific subgroup of raters systematically chooses to use a particular kind of device, it could bias the results. In our case, mobile phones were used by nearly one quarter of raters. The results we report are correlations and we just assume that differences in ratings were influenced by the kind of device raters used. In theory, it is possible that a particular group of raters simultaneously tended to give higher ratings and used mobile phones for viewing and rating. It is likely, however, that the two phenomena are independent of one another, because we found no differences between rater groups in other characteristics.

A potential limitation of our study is the fact that we used a rather specific sample of targets, namely MMA athletes. This fact might limit the generalization of our results. One could expect that MMA fighters would be perceived as highly formidable opponents, as rather specific in appearance (“cauliflower” ears, broken noses, eyebrow scares, etc.), which is why their ratings of formidability and attractiveness might be less variable and/or skewed. In our study, however, raters were not explicitly told that the targets presented to them are MMA fighters. What we found was that mean formidability rating of all three stimuli types on a 7-point scale ≈ 4 (ranging from 2 to 6.2) and skewness of all three stimuli types were between 0.097 and 0.189 (Table 2) and data followed normal distribution. For attractiveness, mean ratings for all three stimuli types were between 2.8 and 2.93 (ranging from 1.7 to 4.8) and skewness between 0.111 and 0.578 (Table 2), hence comparable to average ratings of male facial attractiveness in other studies (e.g., Saribay et al., 2018). It thus seems that the specific nature of our sample does not impede generalization of our finding. Nevertheless, future studies based on less specific samples should further investigate this issue.

To conclude, the findings presented here, along with other recent studies, provide converging evidence that single and multiple view facial images convey highly overlapping information and a single angle view contains enough information about the spatial structural elements of a face to congruently assess formidability and attractiveness, at least in the case of male faces. These results also suggest that studies which use different types of stimulus depiction are, generally speaking, comparable: this ought to simplify the interpretation of individual studies.

## Author Contributions

VT, JF, and JH developed the study concept. KK contributed to the study design. Data collection was performed by VT, JF, DS, and ZŠ. VT performed data analysis and interpretation, VT and JF drafted the manuscript, and DS, ZŠ, KK, and JH provided critical revisions. All authors approved the final version of the manuscript for submission.

### Conflict of Interest Statement

The authors declare that the research was conducted in the absence of any commercial or financial relationships that could be construed as a potential conflict of interest.
