# Supplementary figures and images for: 360 Degrees of Facial Perception: Congruence in Perception of Frontal Portrait, Profile, and Rotation Photographs
Source: Front Psychol. 2018 Dec 7;9:2405. doi: 10.3389/fpsyg.2018.02405 (PMC6293201; doi:10.3389/fpsyg.2018.02405)

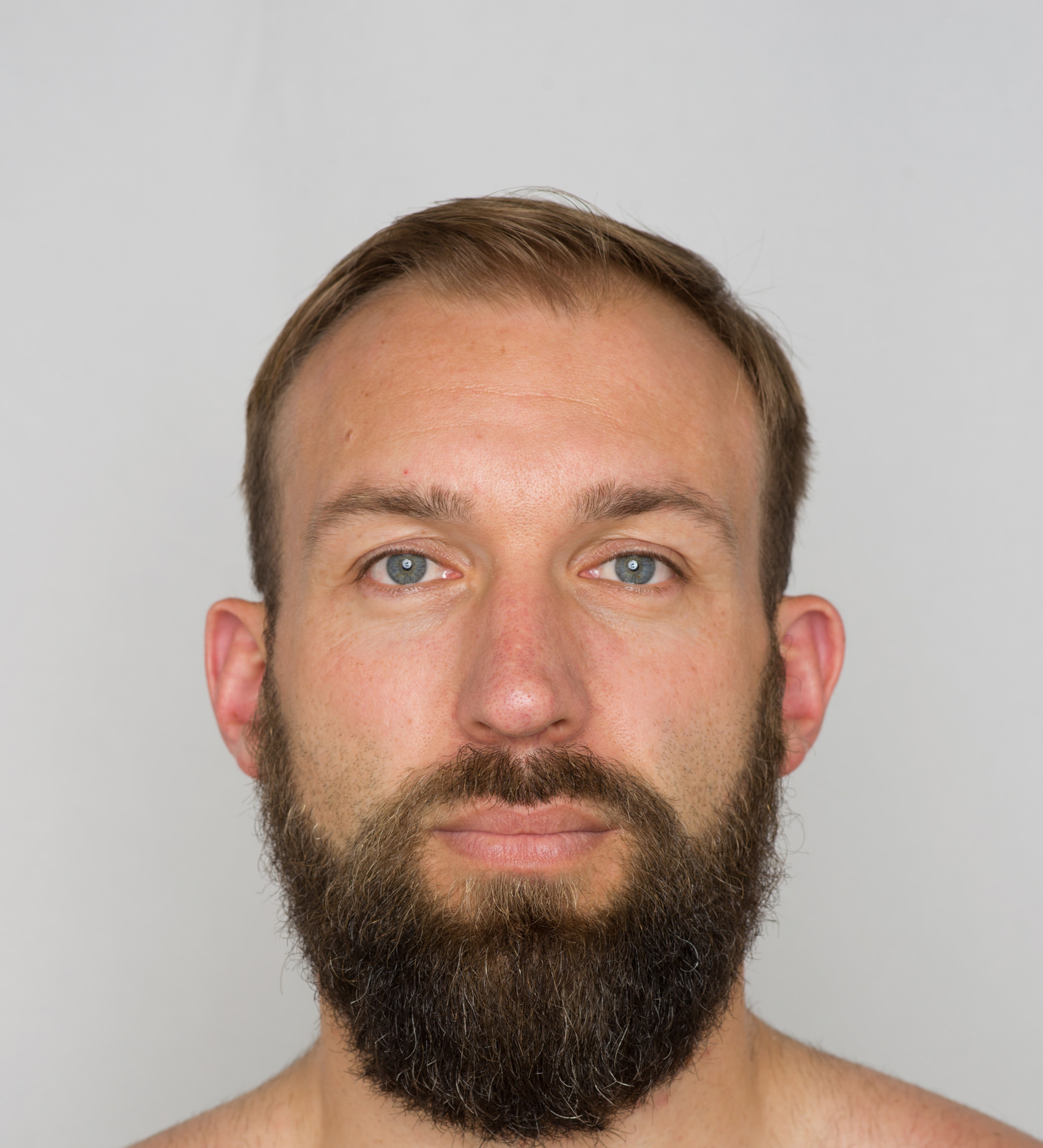

Supplement: Supplementary file 5 [file Image_1.JPEG]

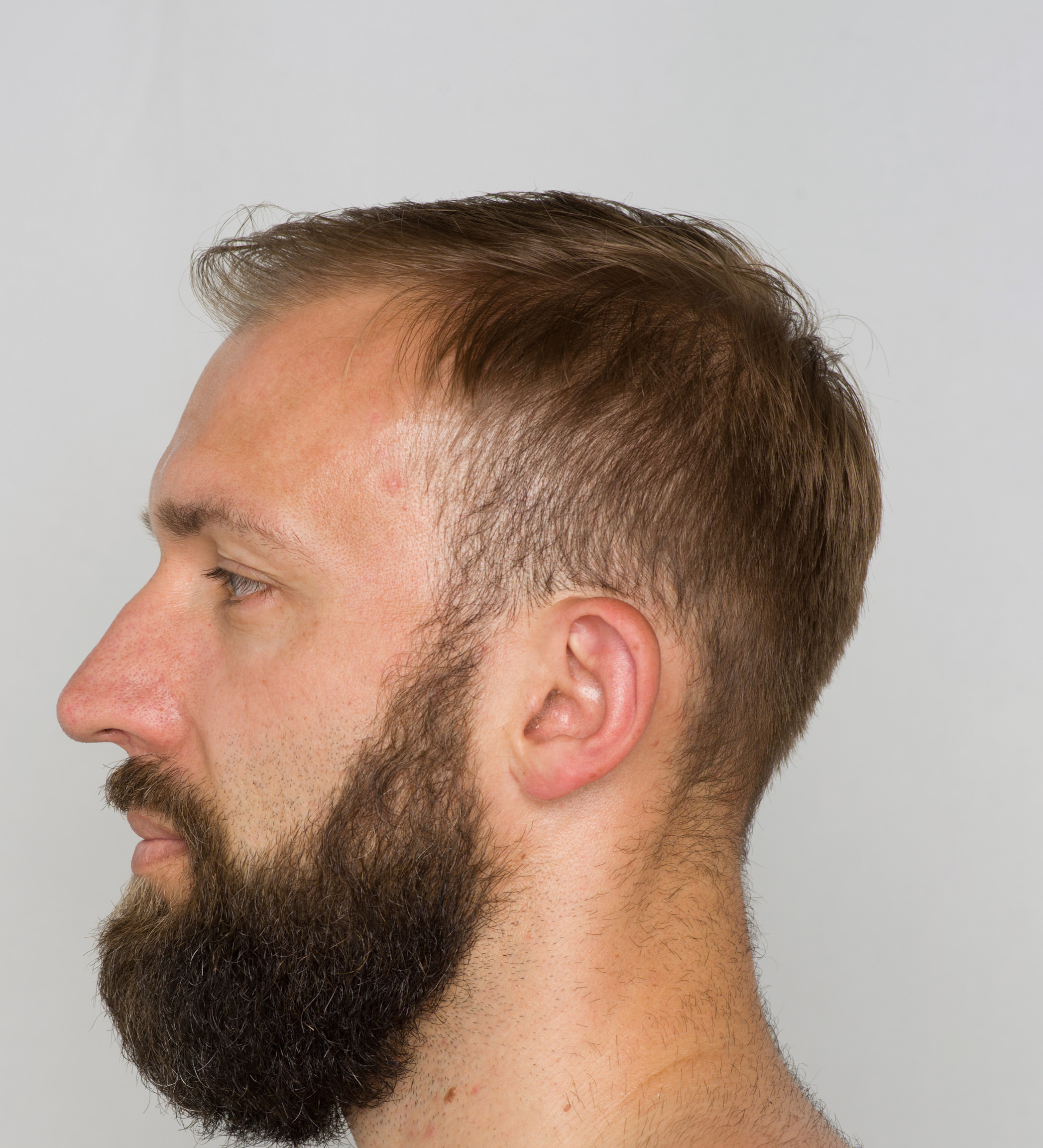

Supplement: Supplementary file 6 [file Image_2.JPEG]
